# A comprehensive assessment of N-terminal signal peptides prediction methods

**DOI:** 10.1186/1471-2105-10-S15-S2

**Published:** 2009-12-03

**Authors:** Khar Heng Choo, Tin Wee Tan, Shoba Ranganathan

**Affiliations:** 1 Institute for Infocomm Research, 1 Fusionopolis Way, #21-01 Connexis, Singapore 138632; 2 Department of Biochemistry, Yong Loo Lin School of Medicine, National University of Singapore, 8 Medical Drive, Singapore 117597; 3 Department of Chemistry and Biomolecular Sciences & ARC Centre of Excellence in Bioinformatics, Macquarie University, Sydney NSW 2109, Australia

## Abstract

**Background:**

Amino-terminal signal peptides (SPs) are short regions that guide the targeting of secretory proteins to the correct subcellular compartments in the cell. They are cleaved off upon the passenger protein reaching its destination. The explosive growth in sequencing technologies has led to the deposition of vast numbers of protein sequences necessitating rapid functional annotation techniques, with subcellular localization being a key feature. Of the myriad software prediction tools developed to automate the task of assigning the SP cleavage site of these new sequences, we review here, the performance and reliability of commonly used SP prediction tools.

**Results:**

The available signal peptide data has been manually curated and organized into three datasets representing eukaryotes, Gram-positive and Gram-negative bacteria. These datasets are used to evaluate thirteen prediction tools that are publicly available. SignalP (both the HMM and ANN versions) maintains consistency and achieves the best overall accuracy in all three benchmarking experiments, ranging from 0.872 to 0.914 although other prediction tools are narrowing the performance gap.

**Conclusion:**

The majority of the tools evaluated in this study encounter no difficulty in discriminating between secretory and non-secretory proteins. The challenge clearly remains with pinpointing the correct SP cleavage site. The composite scoring schemes employed by SignalP may help to explain its accuracy. Prediction task is divided into a number of separate steps, thus allowing each score to tackle a particular aspect of the prediction.

## Background

Signal peptides (SPs) are found at the N-terminus of precursor protein sequences [1]. Prokaryotic and eukaryotic cells utilize these short peptides to mediate the targeting and translocation of the passenger protein domains across the endoplasmic reticulum membrane in eukaryotes or the inner and outer membranes in prokaryotes. SPs are cleaved off from their passenger protein by the endoprotease SPase I [2] upon reaching their targeted destination. In sequence databases such as UniProtKB/Swiss-Prot [3] or EMBL [4], an important annotation task involves the identification of these SPs and the correct identification of their cleavage sites and the start of the mature protein sequences. However, the staggering rate at which unprocessed sequences are being deposited into the sequence databases easily outpaces the results from experimental methods. This has catalyzed the development of faster and more accurate computational methods to automate the task of SP prediction.

SP prediction is fundamentally important as it impacts on other features such as transmembrane topology [5], subcellular localization [6,7], structure modeling and prediction [8], assignment of putative functions to novel proteins and identification of putative cleavage sites in database annotation [9], to name a few examples. Most importantly, the systematic functional annotation of biological sequences using Gene Ontology (GO) [10] requires a precise knowledge of the subcellular localization, where SP prediction has a fundamental input. Some of these prediction tools have been applied with varying degrees of success in genome-wide studies for the discovery of novel secretory proteins or large-scale analyses. Examples include the application in the large-scale Secreted Protein Discovery Initiative (SPDI) which sought to discover novel human secretory and transmembrane proteins in human [11]; identification of secreted proteins in 225 bacterial proteomes [12] and parasitic nematodes [13,14] and genomic analysis of the SARS-associated *Tor2 isolate *coronavirus [15]. Likewise, tools such as SignalP [16] are employed in the annotation of database sequence entries in which experimental evidence is lacking. SP prediction tools can be useful for locating homologous sequences or predicting the correct start codon since SPs are situated at the N-terminal of proteins [17].

Additional file 1 shows a list of SP prediction tools that are publicly available, with the year the tool was first released, methodology and three datasets covered: eukaryotes (Euk), Gram-positive (Gpos) and Gram-negative (Gneg). Earlier reviews [18] and [19] on SP prediction have focussed on comparisons of the machine learning techniques used, rather than evaluating the results of these methods. Except for the two benchmark studies by Meene *et al. *[9] in 2000 and Zhang and Henzel [20] in 2004, which were carried out solely to benchmark the various SP prediction tools available at that time, the majority of the comparison studies were conducted during the development of their respective prediction tool [5,16,17,21-30]. Often, such assessments involved only a subset of the prediction tools that are available or they were tested on a subset of sequences. For instance, the evaluation by Klee and Ellis [31] involved only a subset of the eukaryotic sequences and compared mainly four of the available programs, while Bagos *et al. *[32] evaluated a mix of putative and experimentally verified archaeal SPs. Furthermore, different datasets were used in the evaluation of some of these prediction tools, thus making it extremely difficult to engage in a fair comparison. In some cases, the performance indicators reported actually differ in the aspects that they were investigating (e.g. discrimination of SP or non-SP proteins OR/AND identification of the cleavage site) [28].

The availability of large number of sequences due to the global genome sequencing efforts and the introduction of newer tools (described in Additional file 1) since the previous studies [9,20] have motivated us to conduct a large-scale study to benchmark the gamut of prediction tools. We have carefully collected experimentally verified SPs in a relational database, SPdb [33] (current version 5.1, using SwissProt release 55.0 dated 26 February 2008), with Euk, Gpos and Gneg signal peptide data (see [34] for detailed analysis of SPdb data), suitable for benchmarking prediction tools (see Methods section for details). Using experimentally validated dataset derived from SPdb and Zhang and Henzel [20], we now present a comparison between the different tools that is otherwise often encumbered by the varying accuracies reported in different earlier studies.

## Results

**Figure 1:**
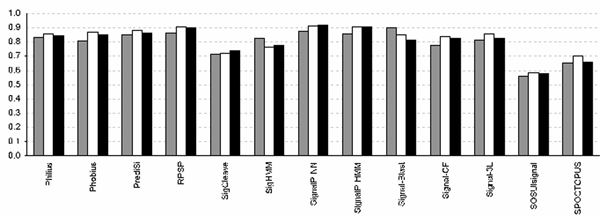
**Aggregated results from all three experiments**. Accuracy results from all three experiments are provided here. For each tool, there are three bars, representing each experiment (gray bar: experiment 1; white bar: experiment 2; black bar: experiment 3).

**Figure 2:**
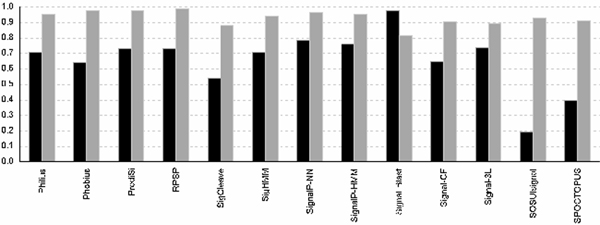
**Results from Experiment 1**. The dataset [20] used in this experiment contains eukaryotic (human) sequences only. The bars colored in light gray represent the specificity while the black bars represent the sensitivity of the prediction tools.

**Figure 3:**
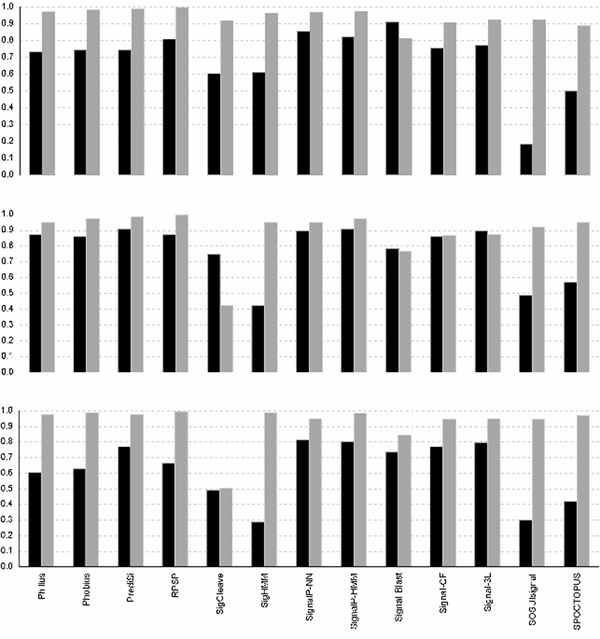
**Results from Experiment 2**. The datasets employed in this experiment are derived from SPdb 5.1 [33] and subjected to manual curation. The datasets are divided into Euk (top chart), Gpos (bottom chart) and Gneg (middle chart) bacteria.

**Figure 4:**
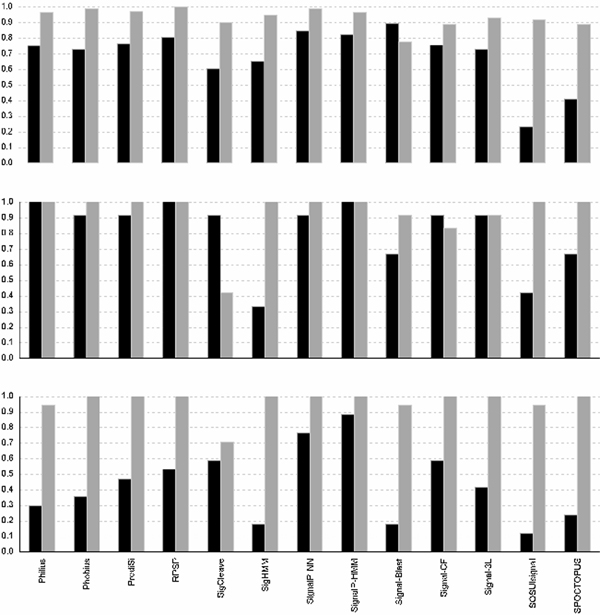
**Results from Experiment 3**. The datasets employed in this experiment are derived from Swiss-Prot Release 57.0 and subjected to the filtering process described in [33]. However, putative SPs which have high probability of existent based on the experiment literature are retained. The datasets are further grouped into Euk (top chart), Gpos (bottom chart) and Gneg (middle chart) bacteria.

To benchmark the 13 SP prediction tools (Additional file 1), we employ our previously developed pipeline [33] to generate 2 datasets that are further curated. An additional dataset containing experimentally verified SPs from Zhang and Henzel [20] is also added to this study. The contents of the datasets are tabulated in Table 1 (the original sequences used to benchmark the tools are provided in Additional file 2). Each dataset is maintained in equal number between the positive and negative instances to ensure that there is no bias in the assessment of the tools. Figures 1, 2, 3, 4 and Table 2 show the results from the three experiments carried out, using the datasets in Table 1 (detailed prediction results for each tool are available from Additional file 3).

**Table 1:** Description of the three datasets developed for benchmarking the thirteen SP prediction tools. Only the first 70aa of the sequence are retained as input. All the negative dataset are subjected to redundancy reduction. T denotes the sequence identity threshold set for redundancy reduction. ^1 ^From a first-pass-filtered set of 9,851 reduced to 4,989 upon redundancy reduction (T = 40%) and atypical/spurious sequences removed; ^2 ^From a first-pass-filtered set of 427 reduced to 230 (T = 40%); ^3 ^From a first-pass-filtered set of 370 reduced to 307 (T = 65%); ^4 ^From a first-pass-filtered set of 8,930 reduced to 4,445 (T = 40%); ^5 ^From a first-pass-filtered set of 110 reduced to 61 (T = 40%); ^6 ^From a first-pass-filtered set of 290 reduced to 150 (T = 40%).

	Dataset for Experiment #1: **Zhang and Henzel **[20] (Experimentally verified SPs)	Dataset for Experiment #2: **SPdb 5.1 **[33] (SPdb 5.1 is derived from Swiss-Prot Release 55.0)	Dataset for Experiment #3: UniProtKB/Swiss-Prot Release 57.0 (excludes datasets used in Experiment #1 and #2)
**Positive**	270 human secreted recombinant proteins	2,349 secretory proteins consisting of:	228 secretory proteins consisting of:
		- Euk: 1874	- Euk: 199
		- Gpos: 168	- Gpos: 17
		- Gneg: 307	- Gneg: 12
**Negative**	270 human non-secretory proteins extracted from SigHMM [26] dataset which is in turn derived from Swiss-Prot Release 40.0.	2,349 non-secretory proteins	228 non-secretory proteins
		- Euk: 1874 (Cytoplasmic and nuclear)^1^	Euk: 199 (Cytoplasmic and nuclear)^4^
		- Gpos: 168 (all cytoplasmic)^2^	- Gpos: 17 (all cytoplasmic)^5^
		- Gneg: 307 (all cytoplasmic)^3^	- Gneg: 12 (all cytoplasmic)^6^

**Table 2:** Benchmark results of the thirteen prediction tools. Equations 1-4 are used to measure the predictive performance of these tools. (Abbreviations used: Sn = Sensitivity; Spc = Specificity; Acc = Accuracy; MCC = Matthews' Correlation Coefficient). ^1 ^Used with HMMER 2.3.2 with cut-off score set at -5 [26] and the updated model [20]; ^2 ^Version 3.0; ^3 ^Authors updated system with UniProt 14.6 (Swiss-Prot Release 57.0) [3,62]; ^4 ^Version 1.0.1.

	Experiment 1				Experiment 2				Experiment 3			
Methods	Sn	Spc	Acc	MCC	Sn	Spc	Acc	MCC	Sn	Spc	Acc	MCC
Philius	0.704	0.952	0.828	0.677	0.742	0.968	0.855	0.729	0.728	0.961	0.844	0.708
Phobius	0.637	0.978	0.807	0.654	0.749	0.982	0.865	0.752	0.711	0.987	0.849	0.726
PrediSi	0.726	0.974	0.850	0.723	0.768	0.986	0.877	0.773	0.750	0.974	0.862	0.742
RPSP	0.730	0.989	0.859	0.744	0.805	0.996	0.901	0.816	0.794	1.000	0.897	0.811
SigCleave	0.541	0.878	0.709	0.445	0.613	0.823	0.718	0.446	0.618	0.860	0.739	0.493
SigHMM^1^	0.707	0.937	0.822	0.662	0.561	0.963	0.762	0.572	0.596	0.952	0.774	0.587
SignalP^2 ^ANN	0.785	0.959	0.872	0.756	0.856	0.965	0.910	0.826	0.842	0.987	0.914	0.838
SignalP^2 ^HMM	0.759	0.952	0.856	0.725	0.832	0.974	0.903	0.814	0.833	0.969	0.901	0.810
Signal-BLAST^3^	0.978	0.815	0.896	0.803	0.881	0.809	0.845	0.692	0.825	0.794	0.809	0.619
Signal-CF	0.648	0.900	0.774	0.566	0.768	0.905	0.836	0.679	0.750	0.890	0.820	0.647
Signal-3L	0.737	0.889	0.813	0.633	0.786	0.920	0.853	0.712	0.715	0.934	0.825	0.665
SOSUIsignal	0.189	0.926	0.557	0.170	0.232	0.925	0.578	0.217	0.232	0.921	0.577	0.212
SPOCTOCUS^4^	0.393	0.907	0.650	0.350	0.502	0.902	0.702	0.441	0.408	0.899	0.654	0.352

### Overall results

Figure 1 depicts the overall accuracy values for all the methods across the three experiments. Experiment 2 and 3 provide values for all three organism groups while Experiment 1 essentially measures the accuracy for Euk alone.

Across the three experiments, SignalP is clearly the most accurate; with the ANN version [16] achieving slightly better results over the HMM version [17]. This is followed by Rapid Prediction of Signal Peptides (RPSP) [24]. It can be seen that most tools achieve accuracies well over 80%, which is consistent with what have been reported in many earlier studies, without complete details of specificity and sensitivity. A breakdown of the prediction results measured by sensitivity and specificity for each experiment, give us a better account of the strength and weakness of each tool.

### Results from experiment 1

The first experiment uses 270 eukaryotic (human) sequences with experimentally verified SPs, from the study by Zhang and Henzel [20].

Based on the results from this experiment (Figure 2 and Table 2), Signal-BLAST predicts the highest number of correct positive instances (i.e. best sensitivity) (0.978). This is dramatically reversed when it scores 0.815 in specificity upon tested with negative instances where it is tasked to distinguish between secretory and non-secretory proteins. This contrasting result is expected since Signal-BLAST which uses a pairwise alignment algorithm (BLAST tool [35]) at its core, needs to find a delicate balance between the two types of datasets in order to achieve a good discrimination. SignalP scores the second best accuracy with the artificial neural network (ANN) version (Acc:0.872; Sn:0.785; Spc:0.959) marginally outperforming the hidden Markov model (HMM) version (Acc:0.856; Sn:0.759; Spc:0.952).

Signal-CF [27] and Signal3L [29] which adopt the "subsite-coupled model" achieve accuracies of 0.774 and 0.813 respectively. The results are lower than those reported in the authors' publications using the same dataset. Manual inspection of Signal-3L revealed that there was a mistake quoted by the authors in their publication [29]. For the entry [Swiss-Prot: Q6UXL0], the authors reported the cleavage site as 28aa instead of the correct 29aa that the authors indicated in their supplied supplementary data ("Online Supporting Information B: Signal-CF dataset - supp-B.txt"). Thus, the tools that were evaluated may have been wrongly penalized (SignalP (version 3.0) and PrediSi [22]). From our examination, Signal-CF and Signal-3L identify the cleavage site at 63aa and 28aa respectively based on the input sequence of length 70aa. When we reduced its evaluation length to LENGTH(SP)+LENGTH(30aa of the mature peptide) which is 59aa in length (the sequence being: MQTFTMVLEEIWTSLFMWFFYALIPCLLTDEVAILPAPQNLSVLSTNMKHLLMWSPVIA) as reported in their publication, Signal-CF and Signal-3L reported SPs of 29aa and 28aa. Comparing the two tools, we noted that selecting the correct "species" option in Signal-3L is critical; otherwise a markedly different length of SP is reported. Signal-CF, on the other hand, is extremely sensitive to the different lengths. Additionally, it is unclear whether the additional classification of sequences into more specific groups (e.g. plant, human, animal etc.) adopted by Signal-3L is able to generate greater advantage over Signal-CF as we shall see in the other experiments.

Sensitivities of SOSUIsignal (0.189) [28] and SPOCTOPUS [30] (0.393) are not comparable to the other methods. This is possibly because identification of cleavage site may not have been a priority in their study [28] as SOSUIsignal was developed to discriminate SPs from non-SPs containing sequences while SPOCTOPUS was developed as a combined predictor for SPs and membrane protein topology.

Other methods namely Philius, Phobius, PrediSi, SigHMM, RPSP and Signal-3L return accuracies that are above 0.800 or 80%. However, closer examination reveals that although their specificities are impressive, their sensitivities are modest, largely in the range of 0.630 to 0.790.

### Results from experiment 2

The second experiment recruits a much larger dataset consisting of 4,704 sequences that are spilt into positive and negative datasets of equal size. The negative set consists of a mix of Euk cytoplasmic and nuclear sequences. The dataset is further divided into the three organism groups (details available in Table 1).

SignalP-ANN (Acc:0.910) and SignalP-HMM (Acc:0.903) achieve the best overall accuracies. This is closely followed by RPSP (Acc:0.901), an extremely fast prediction tool with excellent specificity in discriminating secretory from non-secretory sequences. The results of SigCleave (Acc:0.718; Sn:0.613; Spc:0.823) are marginally lower than that of SigHMM (Acc:0.762; Sn:0.561; Spc:0.963). When we examine their results further by looking at the individual data groups (Figure 3), in particular within the bacterial datasets, SigHMM obtained lower results in the Gneg (Sn:0.420; Spc:0.948) and Gpos (Sn:0.286; Spc:0.988) datasets compared to the Euk (Sn:0.609; Spc:0.963) dataset. A comparable drop in both measurements for the bacterial datasets is observed in Experiment 3 (cf. next section). This is possibly attributed to the newer bacterial sequences that have become available since the model was constructed. SigCleave experiences a similar fall in performance for the Gneg (Acc:0.585; Sn:0.746; Spc:0.423) and Gpos (Acc:0.494; Sn:0.488; Spc: 0.500) datasets. The other prediction tools generally maintain similar trend as observed in the previous experiment, though their sensitivity values are considerably lower in the Gpos dataset compared to the Gneg and Euk datasets.

### Results from experiment 3

New sequences (details available from Table 1) have been extracted from the Swiss-Prot database release 57.0 (totaling 412,525 entries) resulting in a dataset of 228 positive and 228 negative instances. This dataset represents a fresh challenge for majority of the tools except for Signal-BLAST which has been updated recently with Swiss-Prot Release 56.6. The dataset should give us clues for the performance of various tools given an unseen dataset, despite its somewhat smaller size (particularly for the bacterial sequences). The results are presented in Table 2 and Figure 4.

Here, SignalP (both ANN and HMM versions; with HMM scoring higher than ANN) again presents consistently high results. The sensitivity values for other tools plummet particularly when tested with the Gpos dataset. This drop is particularly acute for Signal-BLAST, despite its more recent update. We checked the distribution of the data but do not note any significant differences compared to the previous two datasets.

## Discussion

This study has evaluated a variety of prediction tools (Additional file 1) that incorporate an impressive range of techniques spanning from simple weight matrices to the more sophisticated approach of machine learning algorithms or artificial intelligence approaches. Machine learning techniques appear to be the most popular methods and they have generally attained better accuracies. It was previously suggested that a non-linear feature may be involved in the recognition of cleavage site [17], which perhaps helps to explain the better accuracy achieved by the machine learning-based techniques.

In the case of alignment-based approaches such as Signal-BLAST and SigHMM, their parameters can be tweaked to be more sensitive in identifying cleavage site, but at the expense of its specificity or vice versa. For instance, when we submit the sequence from human carboxylesterase 2 isoform 1 [GenBank:37622885] to Signal-BLAST, a markedly different entry [Swiss-Prot:ICAM1_HUMAN] (with reported cleavage site of 27) was returned as the top hit with an assigned cleavage site of 19. Such a method generally may not be particularly suitable for detecting sequences that share weak homology, since it is highly dependent on how the tool balances sensitivity with specificity.

The majority of the prediction tools achieve better results for the eukaryotic datasets compared to the bacterial datasets. This is possibly due to the larger data size that is available to build models that are sufficiently adequate to describe the underlying distribution. In general, most tools encounter little difficulty in distinguishing between secretory and non-secretory proteins. This is evident from the high specificity achieved even with the new dataset provided in Experiment 3. Other studies involving discrimination between signal anchors and SPs lead to similar conclusions [17]. The identification of the correct cleavage site clearly remains the challenge. In fact, it was reported that as much as one-third of the putatively assigned cleavage sites was observed to be inaccurate [20].

Overall, SignalP remains the leading tool, and has been rather successful in prediction for all three organism groups across the three experiments. The consistency we observe in SignalP (both ANN and HMM versions) may be attributed to its more complex models and robustness of its method where various scoring schemes are devised to tackle different aspects (including SP-likeness, the probability of a segment containing the cleavage site and so on). Also, the sequence window employed by SignalP are also relatively wider (Euk: [-11,+2] representing eleven residues prior to the cleavage site and two residues after the cleavage site, Gneg: [-21,+2], Gpos: [-15,+2]) compared to other methods, which are usually localized to a few residues flanking the SP cleavage location. The majority of the tools clearly require 'active learning' or regular update to their underlying models to reflect the latest data distribution. This is particular so for alignment-based methods as evident from their steady decline in sensitivity over the course of the three experiments.

## Conclusion

This study has critically evaluated thirteen of the most commonly used prediction tools that are available for testing, using identical test datasets, covering eukaryotic sequences as well as combinations of eukaryotic and bacterial sequences. Most of these tools are able to distinguish secretory and non-secretory proteins with little difficulty, although identifying the correct SP cleavage site remains a challenge. Indeed, some tools are more susceptible to changes in the databases, and they are likely to require regular update to their underlying models to reflect the latest observations for a given set of new sequences. This is particular so for alignment-based and matrix-based methods, where the updates will allow proper tuning of their model parameters. The superior and consistent accuracies of SignalP may be attributed to the multiple scoring functions that are used to tackle the different aspects of the prediction task.

## Methods

### Preparation of datasets

Datasets preparation is a crucial step in the development of prediction tools. Often, due to bias data (e.g. over-representation of certain classes of data which were not subjected to redundancy reduction; omission of certain data points, e.g. due to atypical length), the models constructed may not be sufficiently capable of generalizing to new, unseen data. In other cases, inadvertent use of erroneous data to train the predictive models can lead to poor results when tested with new dataset due to the 'noise' found in the training data. To develop the test sets for this work, we have incorporated several good practices proposed in previous works [7,9,17,24,29,36] with our own [33] to generate the following three datasets:

(i) The positive set consists of 270 secreted recombinant human proteins taken from http://share.gene.com/cleavagesite/index.html[20]. As the original study did not create the negative dataset to test the specificity of the tools, we extract 270 human non-secretory proteins from the dataset [26] which was used to construct SigHMM;

(ii) This dataset is taken from SPdb5.1 [33] which is filtered from Swiss-Prot 55.0 and covers most of the data used to develop the majority of the prediction methods compared here. The dataset is further processed following the protocol described in [33]. There are 2349 positive instances (Euk:1874; Gpos:168; Gneg:307), and this is matched by an equal number of negative instances for each organism group. The negative dataset is a mix of cytoplasmic and nuclear (applicable to Euk only) proteins. Proteins from other subcellular localizations are excluded since it is difficult to state unequivocally whether they are secreted [16]. Similarly, single-pass type II membrane proteins that contain signal anchors are skipped since the majority of the entries are predicted http://www.expasy.org/cgi-bin/lists?annbioch.txt and labelled "Potential". We use the "KW" field, instead of "SUBCELLULAR LOCATION" phrase under the "CC" field, to locate the cellular localization due to its more succinct description. Organellar proteins and proteins containing chloroplast or mitochondria transit peptides are also removed. Additionally, entries with the keyword "Secreted" appearing under the "KW" field are removed (e.g. [Swiss-Prot:F13A_HUMAN] which is cytoplasmic in most tissues, but it is secreted in the blood plasma as well). Finally, visual inspection is conducted to remove atypical sequences which consists of only Ms and Qs in its sequence (e.g. [Swiss-Prot:ATX8_HUMAN]). Sequences with SPs that are shorter/longer than the average in the positive set are not excluded, since such sequences do exist and they have been annotated and verified.

(iii) A new set of sequences is extracted from Swiss-Prot Release 57.0 following the protocol described in [33] and in (ii). Sequences (both positive and negative) which are present in (ii) are deliberately omitted (based on their Swiss-Prot *ID *and *accession number*) from this dataset to create a new dataset that is novel for the majority of the tools (except those that have been recently updated such as Signal-BLAST). This would minimize any advantage enjoyed by the tools in predicting SPs from sequences similar to those 'seen' before. Manual inspection of the preliminary filtered set reveals that many of the entries are putative despite the lack of indication in their annotations. Unlike the previous datasets, we are unable to comply with the filtering criteria [33] as it would eliminate 50% of eukaryotic instances and more than 90% of the bacterial sequences. Instead, putative SPs with high probability of existence upon consulting the accompanied literature are retained. However, entries with discrepancies in their report on the cleavage site from Swiss-Prot and the literature such as [Swiss-Prot:CEAM5_HUMAN] and [Swiss-Prot:FAS1_SCHAM], are removed, totalling fourteen eukaryotic sequences. Additionally, two entries which do not have any accompanying experimental literature have been excluded ([Swiss-Prot:A1BG_BOVIN] and [Swiss-Prot:OMPC_GLUDA]).

In all three datasets (both positive and negative sets), the general criteria that we applied to determine the removal of an entry are:

a) Annotation hinting of uncertainty or experimentally unverified (e.g. "probable", "missing", "by similarity", "inferred", "potential", "putative" and "conflict")

b) Lipoprotein cleaved by SPase II ("PROKAR_LIPOPROTEIN" under the "DR" field)

c) Fragment sequence

d) Organellar protein (under "OG" field)

e) *Mollicutes*, a division of bacteria that lack cell wall (under "OC" field)

f) Bacteria without any classification (e.g. [Swiss-Prot:SAT_RIFPS])

g) Sequences with ambiguous characters or non-standard amino acid code (e.g. "X", "Z", "U" etc.) (e.g. [Swiss-Prot:KV3A6_MOUSE])

Duplicates are removed from the positive datasets while negative datasets (non-secretory proteins) are subjected to redundancy reduction using CD-HIT (version 3.1.1) [37] to create a diverse set of sequences. Whenever possible (either bounded by the minimal number of sequences for testing or the lowest CD-HIT threshold that can be set), we adopt the lowest possible threshold.

The popular datasets [9,38] are not adopted in this evaluation since they are derived from earlier Swiss-Prot releases (Release 27.0 and Release 38.0 respectively). Our datasets (Swiss-Prot Release 55.0 onwards) are inclusive of these entries and erroneous entries which were described previously [33] have been manually removed in our datasets.

### Omission of prediction tools

A number of methods that are unavailable for testing are omitted from this study. They include several neural network-based approaches [39,40]; SVMs-based approaches [41-44]; a profile HMM-based method called CJ-SPHMM [45]; matrix-based approach that uses the concept of information theory [46]; a BLOMAP-encoding scheme to transform input sequences [47]; a hybrid approach that uses bio-basis function NNs and decision trees [48]; a global alignment approach based on the Needleman-Wunsch algorithm [49,50] and several earlier prediction tools [51,52]. Other tools such as those for the prediction of subcellular localizations (e.g. *iPSORT *[53], *ProteinProwler *[54] and N-terminus targeting signals (e.g. *Predotar *[55]), that predict the presence of SPs but do not indicate the cleavage sites are excluded as well. We have also omitted specialized tools such as *SecretomeP *which predict non-classical SPs i.e. signal sequences that remain uncleaved [56] and TargetP [57], since it uses SignalP for SP prediction. SPEPlip [58] does not support large-scale testing while SIG-Pred [59] was unavailable for this study.

### Setup of prediction tools

For *PrediSi *[22], we use the web server instead of the standalone version due to the discrepancy in their results. The standalone version reported numerous inaccurate predictions even for the same input sequence. The prediction results are converted to 0 if the result field "Signal Peptide ?" indicates an "N" otherwise the predicted cleavage site is recorded if a "Y" is shown.

For tools which use different models/matrices in their prediction for different organism group [16,17,24-30], the appropriate matrix is selected accordingly. *Signal-3L*, in particular, allows for six selections: (i) human; (ii) plant; (iii) animal; (iv) gram-positive; (v) gram-negative; (vi) "other-eukaryotic". We use the authors' categorization method as shown in (*Online Supporting Information B*: http://www.csbio.sjtu.edu.cn/bioinf/Signal-3L/Data.htm to classify and select the corresponding matrix for a given input sequence.

For *SigCleave *[25], the default threshold (*-minweight*) of 3.5 is used to filter the results.

For *SigHMM *[26], a returned score below -5 is deemed to indicate a non-secretory sequence, otherwise the cleavage site is reported since the sequence is considered as a secretory protein.

For Signal-Blast [21], the detection mode is set to "SP4 - Only Detect Cleavage Site".

For all other tools not specifically mentioned, we have used their default system settings with no additional parameter changes made except selecting the corresponding organism matrices, where available. All parameters for each tool are maintained the same in all three experiments, and the experiments are carried out on 32-bit Intel-based desktop computers equipped with 2 GB of memory.

### Evaluation of prediction tools

Our objective is to benchmark the thirteen SP prediction tools in their ability to identify the correct cleavage sites based on newly generated datasets. All results from the different tools are standardized to the following:

It should be noted that for the case when the returned value is 0, it is possible that the tool may be unable to predict the cleavage site although they may detect the protein as being secretory (e.g. Signal-BLAST for the entry [Swiss-Prot:IGF2_ONCMY]). In the case of non-secretory proteins, the effect of this assignment is negligible since most prediction tools can discriminate extremely well for non-secretory proteins.

To evaluate the predictive performance of the prediction tools, we compute *sensitivity *(Sn), *specificity *(Spc), *accuracy *(Acc) and Matthews' Correlation Coefficient (MCC) (Matthews, 1975). The equations are given by:

where *Sn *and *Spc *measure the fraction of positive instances and fraction of negative instances respectively which have been correctly predicted. *Acc *computes the fraction of positive and negatives instances predicted correctly. *Mcc *returns a value that is between 1 (perfect prediction) and -1 (inverse prediction) where 0 denotes a random prediction. Briefly, sequences which possess cleavable SPs that are subsequently predicted with the correct cleavage sites are designated as true positives (TP). Those that are predicted with the wrong cleavage sites are treated as false negatives (FN). Conversely, sequences without cleavable SPs that are predicted with one are classified as false positives (FP) whereas predictions specifying an absence of SP are considered as true negatives (TN).

## Competing interests

The authors declare that they have no competing interests.

## Authors' contributions

KHC and TTW selected the servers and programs to be evaluated, carried out the computational studies and drafted the manuscript. KHC, TTW and SR participated in the design of the study and interpretation of data. SR supervised the overall project and critically revised the manuscript. All authors read and approved the manuscript.

## Note

Other papers from the meeting have been published as part of *BMC Genomics *Volume 10 Supplement 3, 2009: Eighth International Conference on Bioinformatics (InCoB2009): Computational Biology, available online at http://www.biomedcentral.com/1471-2164/10?issue=S3.

## Supplementary Material

Additional file 1 **A list of the publicly available SP prediction tools**. Software tools that are publicly available for the prediction of SPs (includes the detection of SP and its cleavage site) are listed here. Tools that have been discontinued from development or unavailable for testing are omitted. Abbreviations used in this table (HMM = Hidden Markov model; ANN = Artificial neural network; OET-KNN: Optimized evidence-theoretic K nearest neighbour; PWMs = Position weight matrices; SVMs = Support vector machines). Click here for file

Additional file 2 **The three datasets used in this study**. Text files providing the details of the datasets used in this study are provided. Each file is named as follows: X_Y_Z where X is the experiment number (1, 2 or 3); Y is the organism group (Euk, Gneg or Gpos); Z is the type of dataset [P (positive) or N (negative)]. In the positive set, each entry is formatted as Swiss-Prot ID<space>Cleavage site<space>First 70 amino acid residues. In the negative sets, each entry is formatted as Swiss-Prot ID<space>First 70 amino acid residues. Click here for file

Additional file 3 **Detailed prediction results from the thirteen tools evaluated**. Each worksheet of the 14 worksheets is labelled in the format: X_Y_Z where X is the experiment number (1, 2 or 3); Y ss the organism group (Euk, Gneg or Gpos); Z is the type of dataset [P (positive) or N (negative)]. The overall results are listed at the bottom of worksheets with green tabs. Click here for file
