# Supplementary material for: A comprehensive assessment of N-terminal signal peptides prediction methods
Source: BMC Bioinformatics. 2009 Dec 3;10(Suppl 15):S2. doi: 10.1186/1471-2105-10-S15-S2 (PMC2788353; doi:10.1186/1471-2105-10-S15-S2)
Supplement: Additional file 1 — A list of the publicly available SP prediction tools. Software tools that are publicly available for the prediction of SPs (includes the detection of SP and its cleavage site) are listed here. Tools that have been discontinued from development or unavailable for testing are omitted. Abbreviations used in this table (HMM = Hidden Markov model; ANN = Artificial neural network; OET-KNN: Optimized evidence-theoretic K nearest neighbour; PWMs = Position weight matrices; SVMs = Support vector machines). [file 1471-2105-10-S15-S2-S1.doc]

## Additional File 1

## A list of the publicly available SP prediction tools

Software tools that are publicly available for the prediction of SPs (includes the detection of SP and its cleavage site) are listed here. Tools that have been discontinued from development or unavailable for testing are omitted. Abbreviations used in this table (HMM=Hidden Markov model; ANN=Artificial neural network; OET-KNN: Optimized evidence-theoretic K nearest neighbour; PWMs=Position weight matrices; SVMs=Support vector machines).

| **Name** | **Year** | **Method type** | **Dataset division** | **Description (website URL)** |
| --- | --- | --- | --- | --- |
| Philius  [5] | 2008 | Dynamic Bayesian Networks | No division | Inspired by Phobius, this tool is also designed for transmembrane protein topology prediction. It is capable of predicting SPs as well since it incorporates a SP submodel in addition to a transmembrane submodel. Training data from Phobius [23] is used.  (<http://www.yeastrc.org/philius/pages/philius/runPhilius.jsp>) |
| Phobius  [23] | 2004 | HMM | No division | A combined predictor for transmembrane protein topology and SP where the different regions of transmembrane and SP are modelled respectively. It is presumably better at distinguishing between the two. The tool is trained and tested with newly assembled and curated dataset. The authors claimed to have achieved drastic reduction in misclassification as compared to SignalP-HMM [17] (lower false positive but higher false negative rates).  (<http://phobius.sbc.su.se/>) |
| PrediSi  [22] | 2004 | PWMs | Gpos, Gneg, Euk | This Java-based prediction tool uses 3 matrices (Eukaryotes: [-16, +4], Gpos:[-21, +1] and Gneg:[-16, +2]). Data is extracted from UniProtKB/Swiss-Prot [3] Release 42.9 (Euk:2,783; Gpos:236; Gneg:557 sequences). By using a normalized score of between [0, 1], it allows for comparison between the different matrices. It achieves notably better accuracy for the Gneg dataset as compared to the Gpos and eukaryotic data when it is benchmarked against SignalP (HMM and ANN versions).  (<http://www.predisi.de/>) |
| RPSP  [24] | 2008 | ANN | Gpos, Gneg, Euk | This method uses two ANNs with feed-forward, multi-layer architecture and back-propagation learning algorithm. The combined ANN is more accurate than either the ANN solely trained for eukaryotes or prokaryotes. It claims to be capable of rapidly distinguishing SP from non-SP with high accuracy. The accuracy of the identification of cleavage sites is around 73-78%. Dataset is extracted from Swiss-Prot Release 49.4. Only sequences with amino acid at position -1 that appear in these sets: eukaryotes (A,C,G,L,P,Q,S,T) and bacteria (A,G,S,T) are included. (<http://rpsp.bioinfo.pl/>) |
| SigCleave  [25] | 2008 | PWMs | Gpos, Gneg, Euk | One of the simplest approaches used for the prediction of SP cleavage sites. It uses the modified method for the treatment of positions -3 and -1 in the matrix [60]. Two weight matrices are constructed for the positions from -13 to +2: (a) prokaryotes (based on thirty-six aligned sequences) and (b) eukaryotes (based on 161 aligned sequences). Originally developed by Peter Rice in 1989, it has since been modified by Alex Bleasby. It is available as part of the EMBOSS package.  (<http://emboss.sourceforge.net/apps/cvs/emboss/apps/sigcleave.html>) |
| SigHMM  [26] | 2003 | HMM | Human, Mouse | This method uses the popular HMMER package version 2.2 [61] to generate profile HMMs to model the tri-partite regions in SPs following a previous method [17]. Training data is from human while testing data is from mouse; both sets originate from Swiss-Prot Release 40. The method was later updated using HMMER version 2.3.2 and tested with experimentally verified SP datasets [20].  (<http://share.gene.com/zhang.wood.bioinformatics.2003/sighmm/index.html>) |
| SignalP  [16, 17, 38] | 1997 | ANN | Gpos, Gneg, Euk | The most popular tool for SP prediction. Version 1.0 and 3.0 are based on ANNs. Version 3.0 uses the same architecture as Version 1.0 except that the model has been retrained. It uses two networks to recognize windows containing cleavage sites from non-cleavage sites (*C-score*) and another to distinguish windows with SP and non-SP ones (*S-score*). The maximal combined score termed *Y-score* is used to identify the cleavage site. The *S-score* was subsequently replaced by *D-score* in Version 3.0, which is average of mean *S-score* and the maximal *Y-score*. Different window sizes are used in encoding the ANN. |
|  | 1998 | HMM |  | The accuracy of version 2.0 may not be as good as its ANN version, however, this version is better at detecting the presence of SPs and discriminating between SPs and uncleaved signal anchors.  (<http://www.cbs.dtu.dk/services/SignalP/>) |
| Signal-BLAST [21] | 2008 | Pairwise alignment | Gpos, Gneg, Euk | The pairwise local alignment search tool, BLASTP [35] lies at the heart of this approach. Input sequence is queried against two curated datasets simultaneously to determine to which it is likeliest to belong to. The datasets essentially consist of SPs- and non-SPs- containing sets and a ‘signal peptide bias’ is used to calibrate the comparison. This tool should be easier to maintain compared to other approaches.  (<http://sigpep.services.came.sbg.ac.at/signalblast.html>) |
| Signal-CF  [27] | 2007 | OET-KNN  +  Scaled Window/ Subsite coupling/ Fusing | Gpos, Gneg, Euk | This tool consists of a two-layer predictor where a query protein is first identified as secretory or non-secretory (OET-KNN as classifier) before determining its cleavage site if it is a secretory protein by capitalizing on the subsite coupling effects of (-3, -1, +1) along a protein sequence and fuses the results derived from many width-different scaled windows through a voting system to determine the cleavage site. This tool is better at identifying SP cleavage sites and non-secretory of bacterial sequences as evident from its benchmark against SignalP (HMM and ANN versions) and PrediSi using Swiss-Prot Release 50.7.  (<http://www.csbio.sjtu.edu.cn/bioinf/Signal-CF/>) |
| Signal-3L  [29] | 2007 | Similar to Signal-CF | Gpos, Gneg, Human, Plant, Animal, Euk | This tool expands from the original second layer of Signal-CF to two layers thus creating a three-layer predictor to achieve improvement in accuracy. Data used is from Swiss-Prot Release 50.7.  (<http://www.csbio.sjtu.edu.cn/bioinf/Signal-3L/>) |
| SOSUIsignal [28] | 2004 | Indices (include hydrophobicity | Prokaryotes, Euk | A tri-module system where the first module recognizes the hydrophobic segment in the one-hundred residues at the N-terminal. The second module determines if a sequence possess a SP or otherwise by using a *SS-score*. The final module discriminates SPs from signal anchors using a *SP-score*. Datasets are extracted from Swiss-Prot Release 40. |
| SPOCTOPUS [30] | 2008 | NN+  HMM | No division | An extension of the OCTOPUS tool (originally used for transmembrane protein topology prediction) to provide combined prediction of SPs and membrane protein topology. The training data is the compiled dataset from [23].  (<http://octopus.cbr.su.se/index.php>) |
